# Benign Pancreatic Neurofibroma with Malignant Imaging Features: A Case Report and Literature Review

**DOI:** 10.3389/fsurg.2022.874006

**Published:** 2022-04-07

**Authors:** Ling Song, Zhenpeng Jiang, Jian Cui, BinYang Gao, Yan Luo

**Affiliations:** West China Hospital, Sichuan University, Chengdu, China

**Keywords:** pancreas, neurofibroma, ultrasound diagnosis, general surgery, case report

## Abstract

Pancreatic neurofibroma is a very rare benign neurogenic tumor unrelated to neurofibromatosis type 1 (NF-1). As the volume increases, it has the risk of malignant transformation. The surgical prognosis of pancreatic neurofibroma is good, but its preoperative imaging features are very similar to those of malignant tumors, which may affect the formulation of treatment plans. This article reports a case of giant neurofibroma of the pancreas with contrast-enhanced ultrasound (CEUS) as one of the diagnostic methods and discusses the tumor’s preoperative clinical features, laboratory examinations, and imaging features.

## Introduction

Pancreatic neurofibroma is a benign tumor derived from nerve cells. In the absence of neurofibromatosis type 1 (NF-I), its malignant potential is very low. Isolated neurofibroma unrelated to NF-I is very rare and is not found until they grow too large to cause serious complications (1). This study focuses on analyzing the imaging data of pancreatic neurofibroma and discussing its imaging features to improve the understanding of the disease. Previously reported cases of neurofibromas were all diagnosed using CT or MRI before surgery (2–5). Here, we report a case of pancreatic neurofibroma with contrast-enhanced ultrasound as one of the diagnostic methods.

## Case Presentation

A 35-year-old male patient had dull pain in his right abdomen for 2 months without obvious cause or medical history. At the local hospital, computed tomography (CT) of the upper abdomen revealed a large mass on the neck of the pancreas. Five days later, he came to the biliary surgery department of our hospital for further treatment. On physical examination after admission, the abdomen was soft, and a slight mass was palpable in the upper abdominal area, with poor mobility and mild tenderness. Laboratory examination results showed that CA19-9 was slightly increased, and the values of other tumor markers were normal. White blood cell count, alanine aminotransferase (ALT) and aspartate aminotransferase (AST) were significantly increased, while other laboratory data, such as amylase, lipase and total bilirubin, were not abnormal.

**Figure 1:**
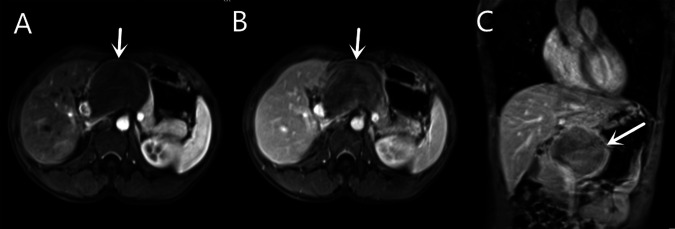
Contrast-enhanced magnetic resonance (MR) of a mass on the pancreatic neck-body. (**A**) T1W Cross-sections MR in arterial phase. (**B**) T1W Cross-sections MR in portal phase. (**C**) T2W Axial MR in arterial phase.

**Figure 2:**
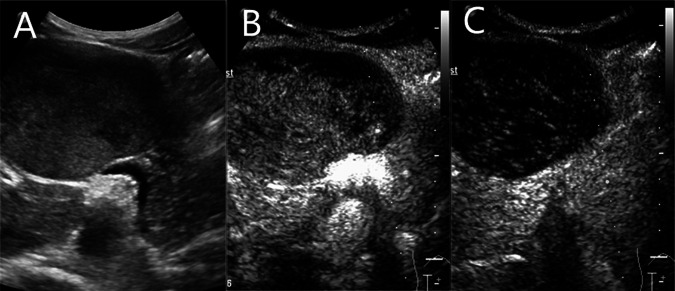
A slightly weaker echogenic mass on ultrasound. (**A**) Two-dimensional ultrasonography. (**B**) contrast-enhanced ultrasound (CEUS) in arterial phase. (**C**) CEUS in parenchymal phase.

Contrast-enhanced magnetic resonance (MR) revealed an 8.2 × 5.7 cm circular confounding signal mass on the pancreatic neck (Figure 1). It had a slightly higher T1 signal and equal T2 signal with a small patch of high T2 signal inside. Contrast enhancement was heterogeneous high enhancement. The adjacent common hepatic artery and splenic artery were pushed, causing their lumens to narrow slightly, and their local boundaries with the tumor were not clear. The fat space between the splenic vein and the tumor is not clear. The main pancreatic duct and bile duct were not dilated. MR finally considered pancreatic malignant cystadenoma or solid pseudopapillary tumor (SPT). Two-dimensional ultrasonography revealed a slightly weaker echogenic mass of approximately 8.6 × 6.4 cm in the pancreatic neck body area, with clear boundaries, clear capsules, heterogeneous internal echoes, and small hypoechoic areas (Figure 2A). CEUS showed rapid and high enhancement at the margin and equal enhancement at the interior of the mass in the arterial phase. In the parenchymal phase, the overall enhancement was low. CEUS considered mostly SPT (Figures 2B,C).

Due to the large size of the mass, the clinician decided to perform an exploratory laparotomy. During the operation, a hard mass of approximately 10 × 8 × 9 cm with a capsule was found above the neck of the pancreas and the portal vein. The tumor surrounds surrounding tissues and organs, including the proper hepatic artery, splenic artery, portal vein and left adrenal gland. Based on the intraoperative findings, the surgeon decided to perform total tumor resection.

**Figure 3:**
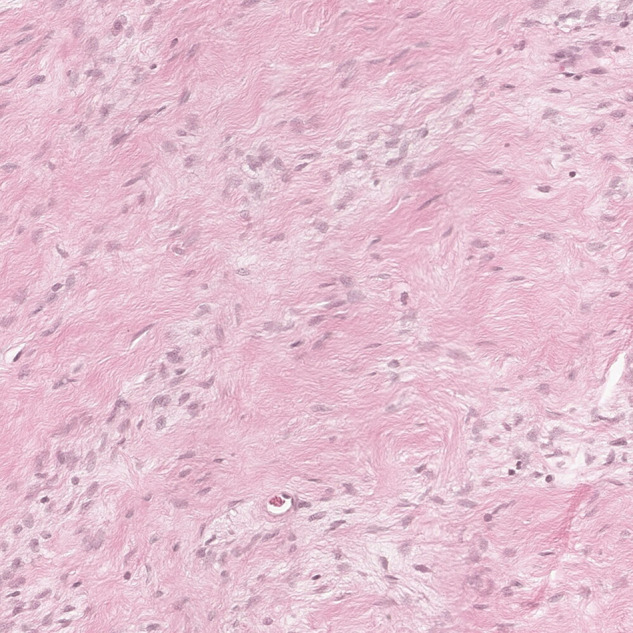
Photomicrograph of pancreatic neurofibroma. Collagen fibers contain a large number of spindle cells (H&E stain, ×100).

**Figure 4:**
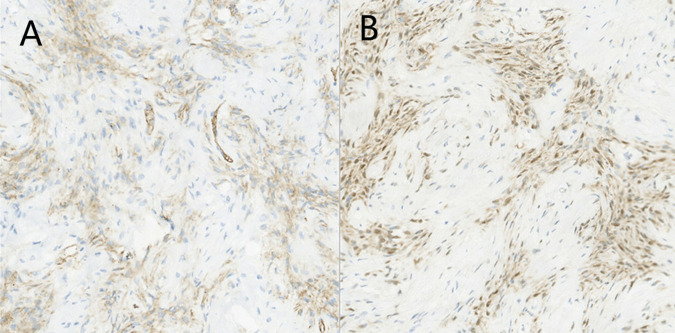
Photomicrograph of pancreatic neurofibroma. (**A**) Immunohistochemical staining [CD34(+)]. (**B**) Immunohistochemical staining [S100(+)].

Histopathology found that the tumor was mainly composed of spindle cells with cytologically bland wavy nuclei in a collagenous matrix (Figure 3). Immunohistochemical staining was S100 (+), CD34 (+), and Ki67 (+2%) (Figures 4A,B). Combined with histological morphology and immunohistochemical results, it was considered a peripheral nerve tumor, and its subtype was consistent with solitary neurofibroma. After two years of follow-up, CT, MRI and CEUS were used alternately for examination, and there was no sign of tumor recurrence Supplementary Materials.

## Discussion

Neurofibroma is a benign neurogenic tumor that can be divided into solitary neurofibroma and neurofibromatosis. Neurofibroma generally originates from peripheral or central nerve cells and is composed of Schwann cells and fibroblasts. Most of them are isolated masses in the dermis or subcutaneous tissue. Neurofibroma occurring locally in the body is the most common, accounting for approximately 90%. It is rare that it appears in the pancreas, and solitary neurofibroma of the pancreas not related to NF-I is even rarer (6).

The solitary neurofibromas that have been reported now mostly appear under the skin, in the bones and in the gastrointestinal tract (3, 5, 7–13). The number of reports of solitary neurofibromas that appeared in the pancreas is very limited (Table 1). Moletta et al. reported that pancreatic neurofibroma combined the cerebral neurofibroma (14). NF-1 was suspected but the patient refused genetic tests. The other three cases were not related to NF-1 (2, 15, 16).

**Table 1:** Clinical characteristics of neurofibromas in the pancreas.

Authors	Year	Age (y)	Gender	Location in the pancreas	Surgical treatment	Association with neurofibromatosis type 1
Moletta et al. (14)	2015	25	Male	Head	Pancreaticoduode-nectomy	Suspicious
Tsai et al. (2)	2012	44	Female	Body	Distal pancreatectomy	No
Imai et al. (15)	1989	57	Male	Uncinate process	Pancreaticoduode-nectomy	No
Kato et al. (16)	1982	51	Male	Head	Total pancreatectomy	No

Unlike superficial neurofibromas, which can be easily palpated, abdominal neurofibromas are most often found because of abdominal pain (2, 17). By this time, the large tumor has already produced a space-occupying effect. Tsai et al. reported that patients with pancreatic neurofibroma had a history of acute pancreatitis, so they believed that the occurrence of solitary neurofibroma might be related to chronic inflammation, ischemia, trauma and other long-term chronic stimulation (2). In this case, the tumor capsule was intact, and the boundary with surrounding tissues was clear. In contrast, the pancreatic neurofibroma we reported was infiltrative growth invading surrounding organs and tissues. Both imaging features and intraoperative findings were very similar to malignant tumors (17).

After the literature review, this study should be the first to report the use of CEUS to participate in the diagnosis and observation of pancreatic neurofibroma. CEUS showed that neurofibroma was a solid masse with signs of fast in and out, which led to the final diagnosis of low-grade SPT. MRI findings of tumor invasion of surrounding tissue made the diagnosis result more inclined to malignant pancreatic cystadenoma. Because the results of the two imaging results were inconsistent and the patient requested surgery, the clinician finally decided to open the abdominal exploration. Because pancreatic neurofibroma is very rare and there is a lack of large-scale research to statistically analyze its imaging characteristics and because the growth pattern of this tumor is very similar to that of malignant tumors, there is indeed a great possibility of misdiagnosis in clinical practice. Histopathology showed that the tumor was mainly composed of spindle cells, and the immunohistochemical results showed that the tumor expressed S-100 protein and CD34 protein, which was consistent with other reports (6, 18).

In the diagnosis process, pancreatic neurofibroma needs to be differentiated from pancreatic mucinous cystic neoplasms and SPT. Unlike pancreatic cystic tumors, pancreatic neurofibroma has little or no cystic components and is not connected to the main pancreatic duct, so there is no dilation of the main pancreatic duct (19). Moreover, although giant pancreatic neurofibroma infiltrates other organs, rarely causes to cause lymph node metastasis or other organ metastasis, such as pancreatic mucinous cystadenocarcinoma (20).

At present, the preoperative differential diagnosis of pancreatic neurofibroma and other pancreatic tumors is still difficult. In clinical practice, various imaging methods should be combined to fully understand the relationship between the tumor and surrounding tissues, and pathological biopsy should be taken to confirm the diagnosis if necessary. Radical resection is often used to treat pancreatic neurofibroma. Most patients have no recurrence or metastasis after surgery, which confirms the effectiveness of surgical resection. It should be noted that the clinician must confirm that it is not related to NF-1 after the diagnosis of neurofibroma (21). The patient had no family history of NF-1 and no symptoms such as café-au-lait spots and subcutaneous nodules, so he was ruled out.

We described the appearance of pancreatic neurofibroma under contrast-enhanced ultrasound, which accumulated experience for the diagnosis of this rare case in the future.

## Conclusion

Pancreatic neurofibroma is a very rare benign tumor of the pancreas. It is very important to fully analyze the imaging data and adopt an individualized surgical approach for patients to preserve the function of the pancreas to the greatest extent and minimize postoperative complications while ensuring complete tumor resection.

## Data Availability

The original contributions presented in the study are included in the article/supplementary material, further inquiries can be directed to the corresponding author/s.
